# Association between dietary selenium intake and the prevalence of osteoporosis and its role in the treatment of glucocorticoid-induced osteoporosis

**DOI:** 10.1186/s13018-023-04276-5

**Published:** 2023-11-15

**Authors:** Yi Luo, Yaolin Xiang, Banghua Lu, Xiaoyan Tan, Yanqiong Li, HuiHui Mao, Qin Huang

**Affiliations:** 1 Department of Nephropathy and Rheumatology, The Central Hospital of Enshi Tujia and Miao Autonomous Prefecture, No. 158, Wuyang Avenue, Enshi City, 445099 Hubei Province China; 2 Department of Neonatology, Renmin Hospital Affiliated to Hubei University for Nationalities, Enshi City, 445099 Hubei Province China

## Abstract

**Background:**

Long-term glucocorticoid therapy may lead to osteoporosis (OP). Selenium (Se) is an essential microelement for human health and bone health. This study evaluated the association between dietary Se intake and the prevalence of OP and further explored the potential therapeutic effect of Se on glucocorticoid-induced OP (GIOP) in vivo and in vitro.

**Methods:**

Data were collected from a population-based cross-sectional study conducted in our hospital. OP is diagnosed based on bone mineral density (BMD) measurements using compact radiographic absorptiometry. Dietary Se intake was assessed using a semi-quantitative food frequency questionnaire. The association between dietary Se intake and OP prevalence was analyzed by multivariable logistic regression. In animal experiments, male Sprague–Dawley rats were intramuscularly injected with dexamethasone (1 mg/kg) daily to induce GIOP, while different doses of Se were supplemented in rat drinking water for 60 d. BMD and biomechanical parameters of rat femur were measured. The histopathological changes of the femur were observed by HE staining, the number of osteoclasts was observed by TRAP staining, and OCN positive expression was detected by immunohistochemical staining. OPG, RANKL, Runx2, and BMP2 in rat femur were detected by Western blot. Bone turnover markers and oxidative stress markers were measured using commercial kits. MC3T3-E1 cells were induced to osteogenic differentiation, stimulated with DXM (100 μM), and/or treated with Se at different doses. Cell proliferation and apoptosis were assessed by CCK-8 and flow cytometry. ALP activity was detected by ALP staining and cell mineralization was observed by alizarin red staining.

**Results:**

Participants with lower dietary Se intake had higher OP prevalence. Se supplementation improved BMD, biomechanical parameters, and histopathological changes of the femur in GIOP rats. Se supplementation also suppressed DXM-induced changes in bone turnover- and oxidative stress-related markers. Under DXM conditions, Se treatment induced MC3T3-E1 cell proliferation, ALP activity, and mineralization.

**Conclusion:**

Lower Dietary Se intake is associated with OP prevalence. Moreover, Se takes a position in bone protection and anti-oxidative stress in GIOP models. Therefore, Se may be a complementary potential treatment for GIOP.

## Introduction

Glucocorticoids (GCs) have anti-immune and anti-infection effects during disease treatments [1, 2]. Prolonged GC treatment commonly causes osteoporosis (OP), resulting in femoral head necrosis and fracture [3–6]. It is recommended that for the treatment of chronic GC, patients should also receive OP-controlled therapies [7–10]. Therefore, drugs to reduce GCs-induced osteoporosis (GIOP) are of great clinical significance.

Selenium (Se), a microelement in the human body, is a component of Se-dependent antioxidant enzymes [10, 11], which can eliminate intracellular reactive oxygen species (ROS) [12, 13], thus regulating cellular processes. Therefore, a lack of Se can lead to increased ROS, which is considered to be the proximal culprit of OP [14]. Several human reports have investigated the relationship between Se levels and bone mineral density (BMD) or osteoporotic hip fractures, but the results have been inconclusive. For example, a cross-sectional study of middle-aged and elderly women found no association between dietary Se intake and BMD [15]. However, the problem is that the majority of the participants were women with normal BMD, meaning that a correlation, if any, between dietary Se intake and low BMD is overlooked. Another cross-sectional study verifies an inverted U-shaped trend between dietary Se intake and BMD [16]. However, the association between dietary Se intake and OP risk is not evaluated due to limitations in the original data. Two other reports indicate a negative correlation between dietary Se intake and OP-induced hip fracture [17, 18]. Other reports have declared a positive correlation between plasma Se concentration and BMD in healthy older European men [19] and healthy postmenopausal women with normal thyroid function [20]. Arachidonate 12-lipoxygenase (ALOX12) variants and serum Se deficiency contribute to OP [21]. But those studies focused on blood Se concentrations, not dietary Se intake.

To fill this knowledge gap, we conducted a cross-sectional study to elaborate on the association between dietary Se intake and OP prevalence. In addition, the biological mechanism of the effect of dietary Se intake on bone health remains uncertain. Therefore, DXM was injected into rats to establish the GIOP model, and MC3T3-E1 cells were cultured in vitro and treated with DXM to simulate the GIOP model, so as to verify the bone protection and anti-oxidative stress effects of Se in vivo and in vitro GIOP models.

## Methods

### Part 1: population cross-sectional study

#### Study population and OP diagnosis

Subjects who underwent health screening in The Central Hospital of Enshi Tujia and Miao Autonomous Prefecture were recruited for this study. All subjects were interviewed regarding a semi-quantitative Food Frequency Questionnaire (SFFQ). The study proposal was approved by the Ethics Committee of The Central Hospital of Enshi Tujia and Miao Autonomous Prefecture, and informed consent from all participants was obtained.

Inclusion criteria: (1) Data retrieval on average consumption of specific foods and beverages over the previous 12 months based on SFFQ; (2) Complete basic information; (3) No musculoskeletal diseases. Eventually, 1200 subjects were included for analysis.

The Alara MetriScan (Alara Inc., USA) compact radiographic absorptiometry (RA) detects BMD in the middle phalanges of the second through fourth fingers of the non-dominant hand. All participants were asked to remove the accessories from their hands before the test to ensure the accuracy of the measurements. The radiographic absorptiometry (RA) system captures a high-resolution radiographic image based on an intensity expressed in any unit (mineral mass/area) on an average value. BMD was compared with the average BMD of healthy subjects of the same gender using a T-score, referring to a database provided by the manufacturer [22, 23]. The external precision measurement system has characteristics of low X-ray dose (< 0.02 μSV/time), low cost, and high portability. Participants were classified according to World Health Organization recommendations. BMD levels are considered normal within 1 standard deviation (SD) compared with healthy subjects. BMD levels lower than 1–2.5 SD are considered osteopenia. BMD levels of 2.5 SD or less are considered OP [24]. Both normal and osteopenic participants were considered non-OP.

#### Assessment of dietary Se intake

In some previously published studies [25, 26], a validated SFFQ was used to assess dietary Se intake. Two SFFQs were conducted at least one week apart to compare and assess repeatability. A random subsample (*n =* 200) was then selected to verify SFFQ by comparing the results obtained from the SFFQ with those obtained from the 24-h diet recall method. The verification results show that the overall performance of SFFQ is consistent with previous studies [27, 28].

Sixty kinds of food are included in the SFFQ according to the general eating habits of Hubei Province, China. The aim was to know how often participants consumed each food (i.e., never, once a month, 2–3 times a month, 1–3 times a week, 4–5 times a week, once a day, twice a day, or > 2 times a day) and the average intake per session (< 100 g, 100–200 g, 201–300 g, 301–400 g, 401–500 g, > 500 g). SFFQ includes 60 common local foods, including major sources of dietary Se [29]. It also includes food sources that contain little Se, such as fruits and vegetables [30]. To facilitate participants to make an accurate choice, a picture of the standard weight of the food is provided. The macro- and micronutrient components of all foods were calculated according to the "Chinese Food Ingredients List " (https://wenku.baidu.com/view/3f2b628488eb172ded630b1c59eef8c75fbf9514.html?from=search).

### Part 2: animal experiment

#### Animals

Animal experiments were approved by the ethical Review Committee of The Central Hospital of Enshi Tujia and Miao Autonomous Prefecture, and were conducted following the guidelines for the care and use of experimental animals. Eight-week-old male SD rats weighing 250 ± 10 g (Beijing Hengji Bioengineering Technology Co., Ltd.) were placed in standard laboratory conditions (25 ± 2 ℃, air humidity of 50 ± 10%, light/dark cycles for 12/12 h), with a sufficient supply of food and water.

#### GIOP induction and Se supplementation

The rats were randomly divided into 5 groups (*n =* 6): Sham group, Model group, 0.1 mg Se group, 0.2 mg Se group, and 0.3 mg Se group. Except for Sham group, other groups were intramuscularly injected with dexamethasone (DXM; 1 mg/kg; Sigma-Aldrich) to induce GIOP. At the same time, the rats in 0.1 mg Se, 0.2 mg Se, and 0.3 mg Se groups were supplemented with different doses of Se in drinking water (as 0.1, 0.2, or 0.3 mg/kg; Na_2_SeO_3_ was prepared in 0.9% NaCl solution). Water was force-fed for 60 d. Finally, rats were euthanized by intraperitoneal injection of 3% pentobarbital sodium (120 mg/kg), and serum and femur were obtained.

#### BMD and biomechanics tests

BMD at the proximal end of the left femur was measured using the PIXImus II densitometer (GE Medical Systems, Lunar Division, USA) with dual-energy X-ray absorptiometry (DEXA).

A single-axis electromagnetic servo testing machine (E1100; Instron, High Wycombe, UK) performed three point bending experiments at room temperature. The femur was placed on the sample carrier and kept wet throughout the experiment. The loading speed was adjusted to 5 mm/min, and the loading span was 10 mm. The maximum bending stress, maximum load and elastic modulus were calculated by the load-deformation curve drawn by computer.

#### Micro-CT analysis

Micro-CT diagnosis is finished by Institute of Laboratory Animal Science, Chinese. The scanning was performed by Inveon micro PET/CT manufactured by Siemens (Berlin, Germany; 60 kV, 400 uA). Samples (31.25 um thick) were scanned in a spatial resolution of 10 um. Inveon analysis workstation was applied for 3D reconstruction analysis. micro-CT scan was performed on a 5 cm proximal end of the femurs. After that, the bone volume/total volume (BV/TV), trabecular number (Tb.N), trabecular separation/Spacing (Tb.Sp) and trabecular thickness (Tb.Th) were computed by the workstation.

#### HE staining

The rat femur specimens were placed in 10% neutral paraformaldehyde and fixed at 25℃ for 1 week. Decalcification was performed in 10% disodium ethylenediamine tetraacetate (formulated in DEPC water). After decalcification, the specimens were dehydrated with ethanol, treated with xylene for 2 h, cleared, embedded in paraffin, and cut into 5 μm thick sections along the long axis of the femur. Paraffin sections were dewaxed in xylene and hydrated with decreasing concentration of ethanol. H&E staining was performed (Solarbio, Beijing, China), and the sections were observed under an optical microscope (DP73; Olympus, Japan) at × 200.

#### Tartrate-resistant acid phosphatase (TRAP) staining

Rat femur sections were heated at 37 °C for 1 min using a TRAP staining kit (Whatman plc; GE Healthcare Life Sciences), dehydrated with gradient ethanol, cleared with xylene, sealed with neutral balm, and analysis was performed with NIKON Eclipse Ci microscope equipped with a digital camera. TRAP-positive cells with three or more nuclei were identified as osteoclasts, and the osteoclast number/bone surface (N.Oc/BS) was calculated by Image-pro plus 6.0 (Media Cybernetics, Inc, Rockville, MD) [31].

#### Immunohistochemistry

Rat femur sections were dewaxed, and antigens were retrieved. Then, the sections were incubated with osteocalcin (OCN) primary antibody (1: 200, ab93876; Abcam) overnight at 4 °C. Goat anti-rabbit IgG (1:1,000, ABIN101988; antibodies-online Aachen) labeled with horseradish peroxidase was used for secondary antibody incubation at room temperature for 60 min. 3,3 '-diaminobenzidine was added, and the sections were counterstained with hematoxylin and viewed under an optical microscope (Olympus) at × 200. The known positive film was regarded as the positive control and the first antibody was replaced by phosphate buffered saline (PBS), which served as the negative control. A total of 3 fields were randomly selected from each section and image analysis was performed using ImageJ 6.0 software, OCN positive staining (brown) cells were counted [32].

#### Serum OCN and C-terminal telopeptides of type I collagen (CTX)

Commercial ELISA kits for OCN and CTX (USCN Life Sciences, Wuhan, China) tested serum OCN and CTX. In simple terms, diluted serums were incubated with OCN and CTX antibodies for 1 h and mixed with streptavidin-HRP for 30 min, both at 37 ℃. Absorbance values at 450 nm were recorded on the microplate reader (ELX-800; Bio-Tek Instruments).

### Part 3: In vitro test

#### Cell culture

MC3T3-E1 cells (ATCC, VA, USA) were cultured in DMEM (Gibco, NY, USA) consisting of 10% FBS (Gibco) at 37 °C with 5% CO_2_. The medium was supplemented with β-glycerophosphate (10 mM) and ascorbic acid (50 mg/L) as osteogenic differentiation induction solution, in which MC3T3-E1 cells were treated with 100 μM DXM for 7 d with or without Se (0.5 μM and 1 μM).

#### CCK-8

MC3T3-E1 cells were inoculated in 96-well plates at 5 × 10^3^ cells/well. After DXM and Se treatments, CCK-8 reagent (Sigma-Aldrich) was cultured for 2 h at 10 mL/well. Absorbance at 450 nm was read using a microplate reader (ELX-800, Bio-Tek Instruments).

#### Flow cytometry

Annexin V-FITC Apoptosis Detection Kit (BD Biosciences, USA) analyzed cell apoptosis. MC3T3-E1 cells were incubated in a 6-well plate at 2 × 10^5^ cells/well for 24 h, re-suspended with 200 μL Annexin V-FITC and 10 μL PI, and analyzed on a flow cytometer (BD Biosciences, USA).

#### Malondialdehyde (MDA), glutathione (GSH), and superoxide dismutase (SOD) measurements

According to the manufacturer's instructions, MDA (S0131S; Beyotime, China), GSH (A006-1–1; Nanjing Jiancheng Bioengineering Institute, Nanjing, China), SOD (S0109; Beyotime) content were measured in the supernatant of MC3T3-E1 cell culture medium.

#### Western blot

Total protein was extracted using RIPA buffer (Beyotime, Shanghai, China) and checked for protein concentration based on a BCA kit (Beyotime). Proteins were isolated with 12% SDS-PAGE and transferred to PVDF membranes (Bio-Rad, CA, USA), followed by being blocked with 5% skim milk for 1 h. The membrane containing the target protein was mixed overnight with primary antibody OPG (1:100, CST), RANKL (1:100, Abcam), Runx2 (1:1000, Abcam), BMP2 (1:1000, Abcam), and GAPDH (1:1000, Abcam) at 4 °C, paired with anti-rabbit IgG or anti-mouse IgG secondary antibody (1:2000; ZSBG-Bio, Beijing, China) for 2 h, developed with an enhanced chemiluminescence reagent (Beyotime) and analyzed with Image J software.

#### ALP staining

MC3T3-E1 cells were induced for 7 d in an osteogenic medium, washed twice with PBS, and treated with 4% paraformaldehyde (Solarbio). After 3 rinses with PBS, ALP staining was performed for 30 min using the BCIP/NBT staining kit (Beyotime). After 3 rinses with deionized water, the sample was observed under an optical microscope (Olympus).

#### Alizarin red staining

After 21 d of osteogenic induction, cell mineralization was detected using the alizarin red staining kit (Sigma-Aldrich). MC3T3-E1 cells were stained with 2% alizarin red (pH = 4.2) for 10 min, rinsed with distilled water, and viewed with microscopy (Olympus) to observe mineralized nodules.

#### Statistical analysis

Kolmogorov–Smirnov test evaluates the normality of variables. Analysis of variance and Kruskal–Wallis test verified differences between means, and Chi-square test and Fisher's Exact test analyzed differences between proportions. Post-hoc Tukey and Bonferroni tests assessed statistical differences between groups of parametric and nonparametric variables, respectively. According to the quartile distribution of the study population, dietary Se intake was divided into four quartiles: the first quartile (≤ 29.2 μg/day), the second quartile (29.3–39.8 μg/day), the third quartile (39.9–51.8 μg/day), and the fourth quartile (≥ 51.9 μg/day). Odds ratios and their respective confidence intervals were calculated using the first quartile as a reference. A multivariate linear regression model was used to assess the association between dietary Se intake and OP prevalence. Three models were used in the current analysis: Model 1 was adjusted for dietary energy intake; Model 2 was further adjusted according to age, gender, and BMI based on Model 1. Model 3 was further adjusted according to smoking status, alcohol consumption, diabetes, hypertension, physical activity, dietary calcium and fiber intake based on Model 2. Gender subgroup analysis was then performed. Statistical software SPSS 21.0 and STATA 11.0 were applied for data analysis.

## Results

### Basic characteristics of the subject

A total of 1200 subjects (680 males, 520 females) with a mean age of 50.6 ± 9.2 years were enrolled. OP prevalence was 6.5% (1.6% in males and 12.9% in females). Table 1 shows the basic characteristics of the subjects, finding significant differences in terms of gender, age, smoking and alcohol consumption status, BMI, hypertension, physical activity, and dietary energy, fiber, calcium, and Se intake in OP and non-OP participants.

**Table 1:** Basic characteristics of participants

Basic characteristics	OP population	Non-OP population	*P*
Number	78	1122	
Gender			< 0.001
Male (n, %)	11 (14.1)	669 (59.6)	
Female (n, %)	67 (85.9)	453 (40.4)	
Age (years)	58.5 ± 6.4	50.6 ± 7.2	< 0.001
Smoking (n, %)	7 (9.0)	299 (26.6)	< 0.001
Drinking (n, %)	14 (17.9)	471 (42.0)	< 0.001
BMI (kg/m^2^)	22.8 ± 3.2	24.5 ± 3.0	< 0.001
Diabetes (n, %)	10 (12.8)	124 (11.1)	0.58
Hypertension (n, %)	35 (44.9)	348 (31.0)	0.016
Activity level (h/week)	2.8 ± 2.7	2.1 ± 2.9	0.039
Dietary calcium intake (mg/day)	442.6 ± 180.0	488.3 ± 195.0	0.045
Dietary fiber intake (g/day)	15.4 ± 10.8	18.6 ± 12.2	0.024
Dietary energy intake (Kcal/day)	1480.5 ± 650.0	1630.8 ± 600.0	0.034
Dietary selenium intake (μg/day)	39.2 ± 19.7	45.5 ± 22.6	0.017

Footnote: OP, osteoporosis

### Association between dietary Se intake and OP prevalence

Table 2 indicates the correlation between dietary Se intake and OP prevalence. Model 1 revealed a negative correlation after adjusting for dietary energy intake. Both Model 2 and 3 turned to a negative correlation between dietary Se intake and OP prevalence. Females and males got similar results.

**Table 2:** Association between dietary selenium intake and the prevalence of OP

	Quartiles of dietary selenium intake (μg/day)				*P* for trend
	Q1(≤ 29.2)	Q2 (29.3–39.8)	Q3 (39.9–51.8)	Q4 (≥ 51.9)	
Median selenium intake (μg/day)	22.8	34.6	45.2	63.4	
Total					
Model 1 (95% CI)	1.00 (Ref.)	0.79 (0.62, 1.00)	0.75 (0.55, 1.02)	0.49 (0.33, 0.73)	< 0.001
Model 2 (95% CI)	1.00 (Ref.)	0.73 (0.56, 0.95)	0.71 (0.50, 1.02)	0.45 (0.29, 0.71)	< 0.001
Model 3 (95% CI)	1.00 (Ref.)	0.73 (0.56, 0.96)	0.72 (0.50, 1.04)	0.47 (0.30, 0.74)	< 0.001
Male					
Model 1 (95% CI)	1.00 (Ref.)	0.34 (0.17, 0.67)	0.37 (0.18, 0.78)	0.19 (0.07, 0.45)	< 0.001
Model 2 (95% CI)	1.00 (Ref.)	0.34 (0.17, 0.69)	0.40 (0.19, 0.84)	0.21 (0.08, 0.51)	< 0.001
Model 3 (95% CI)	1.00 (Ref.)	0.36 (0.18, 0.72)	0.41 (0.21, 0.90)	0.24 (0.09, 0.60)	< 0.001
Female					
Model 1 (95% CI)	1.00 (Ref.)	0.78(0.60, 1.01)	0.73 (0.51, 1.06)	0.52 (0.33, 0.83)	< 0.001
Model 2 (95% CI)	1.00 (Ref.)	0.84 (0.63, 1.12)	0.79 (0.53, 1.20)	0.53 (0.32, 0.89)	< 0.001
Model 3 (95% CI)	1.00 (Ref.)	0.83 (0.61, 1.11)	0.78 (0.52, 1.20)	0.52 (0.31, 0.88)	< 0.001

Model 1, adjustment according to dietary energy intake; Model 2, further adjustment according to age, gender and BMI on the basis of Model 1; Model 3, further adjustment according to smoking status, alcohol consumption, diabetes, hypertension, physical activity level, dietary calcium intake and dietary fiber intake on the basis of Model 2

### Effect of Se supplementation on BMD and histopathological changes of femur in GIOP rats

**Fig. 1:**
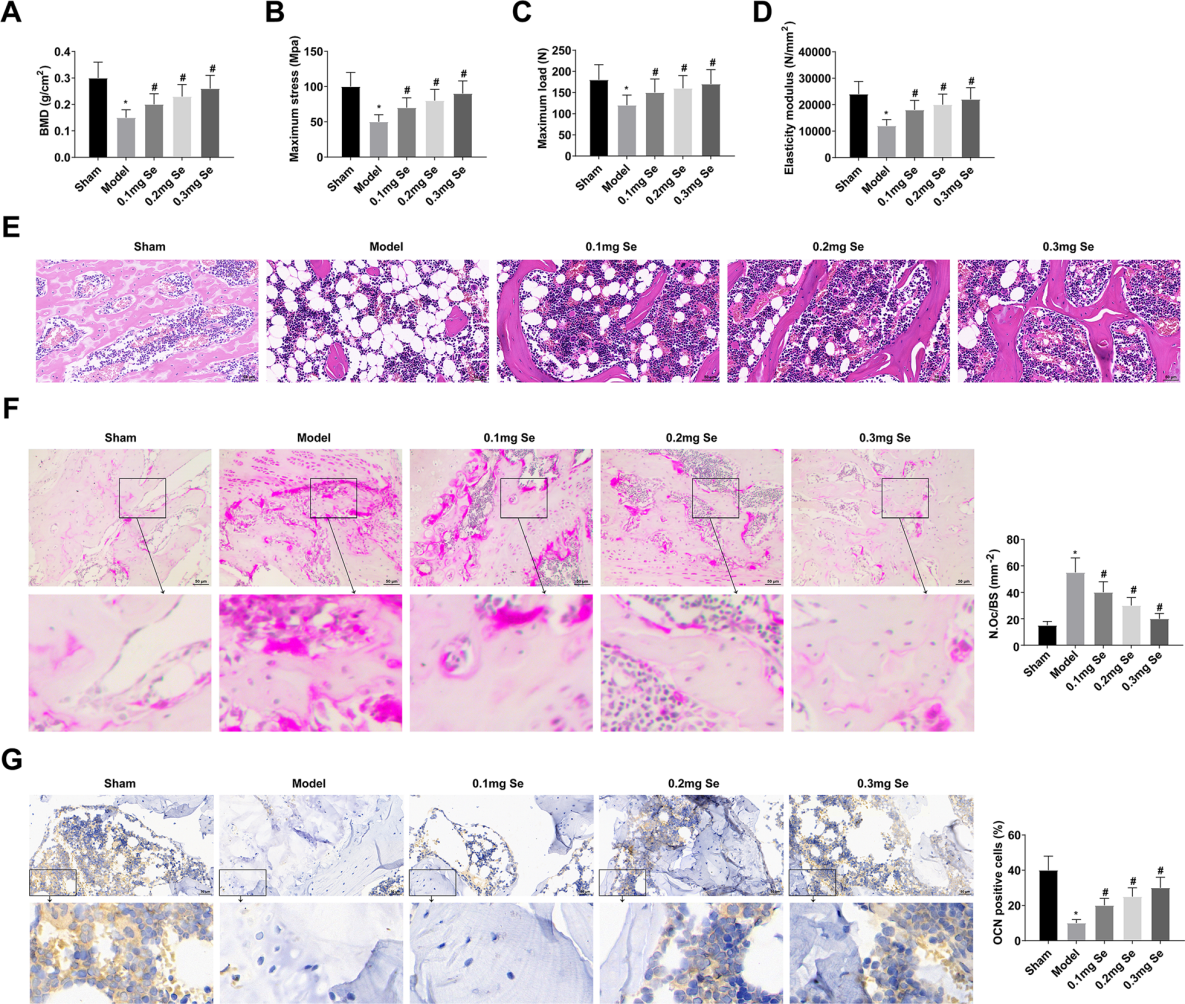
Effect of Se supplementation on BMD and histopathological changes of femur in GIOP rats. **A** BMD was measured by DEXA; **B**–**D** Biomechanical parameters of femur were determined by three-point bending test; **E**–**F** Histopathological changes of femur were observed by HE staining and TRAP staining; **G** Immunohistochemical detection of OCN positive expression. Values are expressed as mean ± SD. *n =* 6; * *vs.* Sham, *P* < 0.05; # *vs.* Model, *P* < 0.05

SD rats were given intramuscular injection of DXM (0.1 mg/kg) once a day to induce GIOP, while different doses of Se (0.1, 0.2 or 0.3 mg/kg) were added to drinking water every day for 60 d. BMD was measured by DEXA. Results showed that femur BMD in GIOP rats was decreased, while Se supplementation improved BMD (Fig. 1A). Biomechanical parameters of the femur were determined by a three-point bending test, manifesting that the maximum stress, maximum load, and elastic modulus of GIOP rats were reduced, while Se supplementation improved these parameters (Fig. 1B–D). In addition, BV/TV, Tb.N, Tb.Sp and Tb.Th were also measured, and the results showed that after DXM treatment, BV/TV, Tb.N and Tb.Sp were reduced, while Tb.Sp was increased, while Se supplementation could increase BV/TV, Tb.N and Tb.Th, and reduce TB.SP (Table 3). Figure 1E shows the vertical metaphyseal of the femur in each group after HE staining. Under light microscopy, trabecular arrangement was tight and regular in Sham group, trabecule was significantly thinner and bone marrow cavity increased in Model group, while after Se supplementation, trabecular thickness was increased and bone marrow cavity decreased. TRAP is a specific marker enzyme of osteoclasts and is distributed in the cytoplasm of osteoclasts. TRAP staining showed fewer osteoclasts in Sham rats, increased osteoclasts in model rats, and osteoclasts were reduced after Se supplementation (Fig. 1F). Immunohistochemical detection of OCN-positive expression showed that there were more OCN-positive cells (osteoblasts) in Sham rats, significantly reduced OCN-positive cells in model rats, and OCN positive cells were increased after Se supplementation (Fig. 1G).

**Table 3:** Changes of micro-CT parameters

	Sham	Model	0.1 mg Se	0.2 mg Se	0.3 mg Se
BV/TV	0.42 ± 0.05	0.25 ± 0.03*	0.31 ± 0.06#	0.34 ± 0.04#	0.36 ± 0.03#
Tb.N (mm^−1^)	4.52 ± 0.40	3.18 ± 0.18*	3.76 ± 0.35#	3.93 ± 0.28#	4.07 ± 0.72#
Tb.Sp (μm)	0.14 ± 0.03	0.37 ± 0.02*	0.20 ± 0.05#	0.17 ± 0.03#	0.15 ± 0.08#
Tb.Th (μm)	0.11 ± 0.04	0.06 ± 0.02*	0.08 ± 0.02#	0.09 ± 0.03#	0.10 ± 0.05#

Data represent the mean ± SD of 6 rats.; * *vs.* Sham, *P* < 0.05; # *vs.* Model, *P* < 0.05

### Effect of Se supplementation on bone metabolism in GIOP rats

**Fig. 2:**
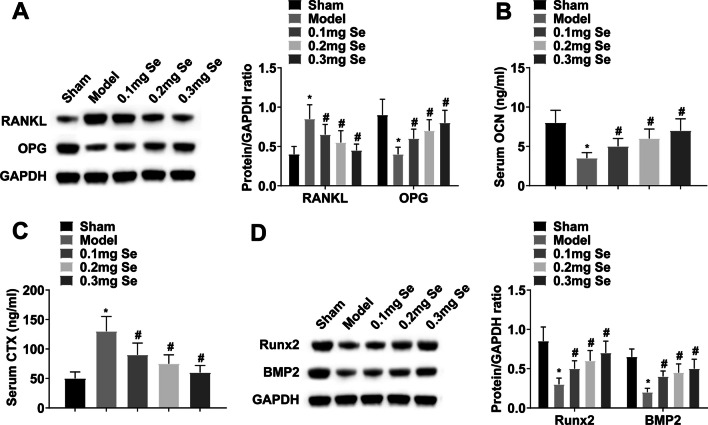
Effect of Se supplementation on bone metabolism in GIOP rats. **A** RANKL/OPG in femur tissue was detected by Western blot; **B**–**C** Serum OCN and CTX levels; **D** Runx2 and BMP2 in femur were detected by Western blot. Values are expressed as mean ± SD. *n =* 6; * *vs.* Sham, *P* < 0.05; # *vs.* Model, *P* < 0.05

RANKL/OPG ratio can reflect bone metabolism. Western blot results showed that DXM induced RANKL and suppressed OPG, while Se supplementation could promote RANKL expression and inhibit OPG expression (Fig. 2A). Serum bone turnover markers (OCN and CTX) of GIOP rats were then measured. OCN levels were lowered and CTX levels were elevated in GIOP rats, while Se supplementation could reverse the changes in OCN and CTX levels (Fig. 2B, C). In addition, Se supplementation offset DXM-induced downregulation of osteogenic factors (Runx2 and BMP2) expression (Fig. 2D).

### Effect of Se supplementation on serum markers of oxidative stress in GIOP rats

**Fig. 3:**
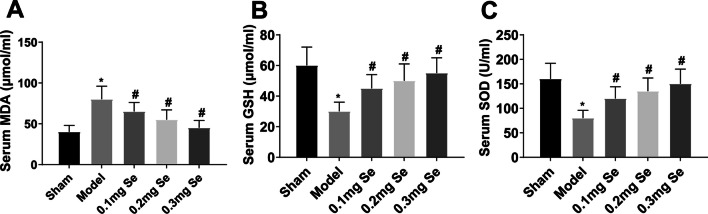
Effect of Se supplementation on serum markers of oxidative stress in GIOP rats. **A**–**C** Levels of serum oxidative stress markers (MDA, GSH and SOD) in GIOP rats. Values are expressed as mean ± SD. *n =* 6; * *vs.* Sham, *P* < 0.05; # *vs.* Model, *P* < 0.05

Serum oxidative stress markers (MDA, GSH, and SOD) were measured in GIOP rats. MDA levels increased while GSH and SOD levels decreased in GIOP rats, and Se supplementation could reverse the changes in MDA, GSH, and SOD (Fig. 3A–C).

### Effects of Se on function and oxidative stress of MC3T3-E1 cells

**Fig. 4:**
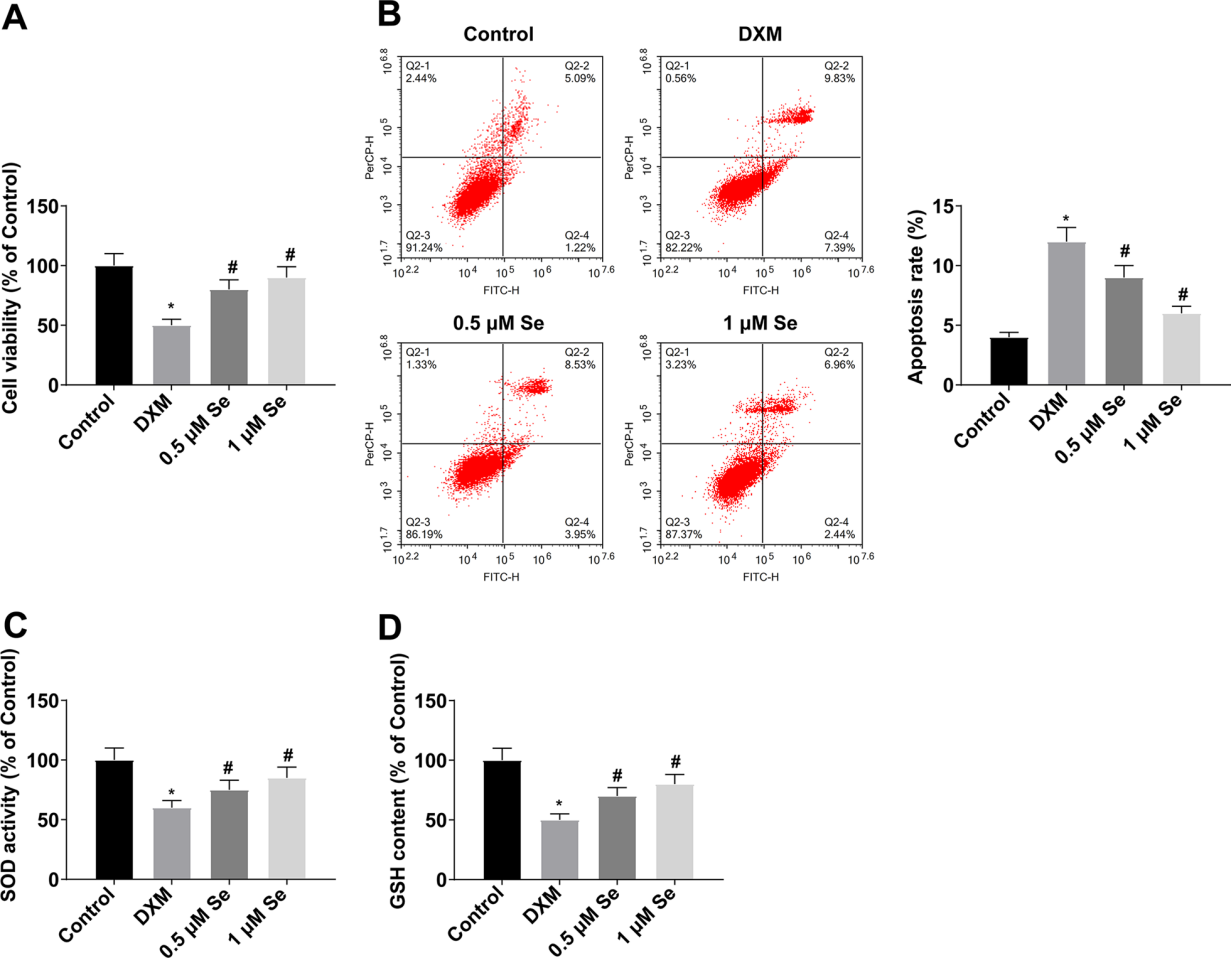
Effects of Se treatment on MC3T3-E1 cell function and oxidative stress. **A** MC3T3-E1 cell proliferation was detected by CCK-8; **B** MC3T3-E1 cell apoptosis was detected by flow cytometry; **C**–**D** Activity of antioxidant enzymes (SOD and GSH) in MC3T3-E1 cells. Values are expressed as mean ± SD; *N =* 3; * *vs.* Control, *P* < 0.05; # *vs.* DXM, *P* < 0.05

To further determine the bone protective effect of Se in GIOP, CCK-8 detected MC3T3-E1 cell proliferation, and flow cytometry detected cell apoptosis. DXM suppressed MC3T3-E1 cell proliferation and enhanced apoptosis, which could be offset by Se treatment (Fig. 4A, B), suggesting that Se treatment had a protective effect on MC3T3-E1 cells. Antioxidant enzymes were analyzed in MC3T3-E1 cells to verify the antioxidant stress effect of Se, and the outcomes reported that Se treatment could activate SOD and GSH activities (Fig. 4C, D).

### Effects of Se treatment on ALP activity and mineralization in MC3T3-E1 cells

**Fig. 5:**
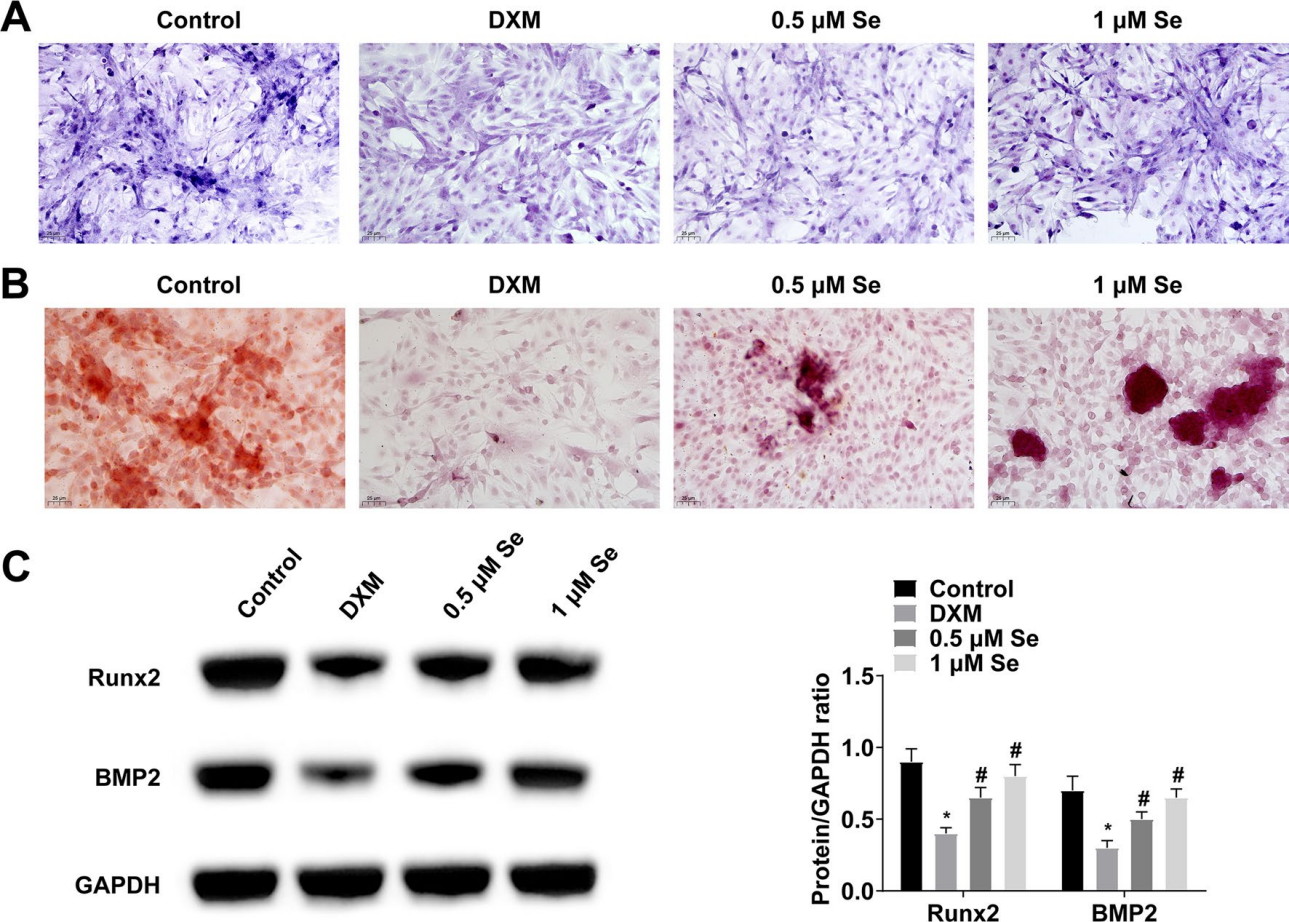
Effect of Se treatment on ALP activity and mineralization in MC3T3-E1 cells. **A** ALP activity was analyzed by ALP staining; **B** Formation of mineralized nodules was observed by Alizarin red staining; **C** Runx2 and BMP2 in MC3T3-E1 cells were detected by Western blot. Values are expressed as mean ± SD; *N =* 3; * vs. Control, *P* < 0.05; # *vs.* DXM, *P* < 0.05

Finally, ALP staining and alizarin red staining analyzed ALP activity and mineralization of MC3T3-E1 cells. It was seen that DXM inhibited ALP activity and mineralization of MC3T3-E1 cells, and Se treatment could attenuate these changes (Fig. 5A, B). In addition, DXM-induced decrease in Runx2 and BMP2 expression was also significantly improved by Se treatment (Fig. 5C).

## Discussion

OP is featured by weak bones and high risk of fracture, which is the main cause of fracture in the elderly population [33–38]. Epidemiological investigation has gradually confirmed that glucocorticoids contribute to the occurrence of secondary OP [39]. Oxidative stress can induce osteoblast damage and is the pathologic mechanism of GIOP, so antioxidants can be utilized for the treatment or prevention of GIOP [40]. Elemental Se is an essential micronutrient that, when combined with protein polypeptide chains, forms selenoproteins, which are involved in many biological mechanisms [41–44]. The proportion of Se is highest in skeletal muscle (27.5%), followed by bone (16%) [45]. Inconsistent epidemiological results have been obtained due to a lack of reports on the association between dietary Se intake and bone health. It has been examined that no improvement of bone loss can be achieved by dietary Se in male breast cancer models [46]. Also, another report indicates that Se intake (200 μg/day) has no effect on bone health in postmenopausal women [47]. These results are different from those of this study, possibly due to experimental errors caused by insufficient sample size. This study found a negative association between dietary Se intake and OP prevalence, which is consistent with the results of a cross-sectional study [48].

Uncertainty has remained concerning the biological mechanism of the effect of dietary Se intake on bone health. Redox status can mediate bone remodeling to sustain bone regeneration through osteocytes/osteoblasts/osteoclasts interwork [49]. ROS can induce osteoblast apoptosis, form osteoclasts, and inhibit mineralization and osteogenesis. Oxidative stress causes an imbalance in osteoclast formation, which increased bone remodeling and bone loss [50, 51]. Selenoprotein mediates the physiological function of Se to exert antioxidant function, maintain Redox balance, and modulate cell proliferation and differentiation [52]. To verify the bone protection and anti-oxidative stress effects of Se, DXM was injected into rats to establish the GIOP model, and MC3T3-E1 cells were treated with DXM to simulate the GIOP model.

In OP, decreased bone mass and deteriorated bone microstructure can be seen [53, 54]. Animals treated with GC have decreased BMD [55, 56]. In this study, DXM led to a significant decrease in BMD, but Se supplementation elevated BMD. In addition, DXM caused the reduction of maximum stress, maximum load, and elastic modulus, while Se supplementation reversed these parameters. The proximal femur is rich in trabecular bone, which is one of the bone types most prone to GCs [57–59]. In this study, the femur structure was damaged after DXM stimuli, manifested as bone lacunae and trabecular thinning, and these injuries were weakened after Se supplementation.

RANKL and OPG are modifiers in bone formation and bone resorption balance in GIOP [60]. RANKL can attach to RANK and then initiate bone resorption by increasing osteoclast activity. In contrast, OPG prevents RANKL-RANK interaction and inhibits bone resorption [61]. RANKL upregulation alters bone homeostasis and ultimately initiates OP [62, 63]. The current study manifested that DXM increased RANKL and decreased OPG levels in GIOP rats, and these changes were attenuated by Se supplementation. OCN is a marker of advanced bone differentiation, and CTX is a marker of bone resorption [64]. Our results showed that DXM caused a decrease in OCN levels and an increase in CTX levels, while Se supplementation attenuated these changes. BMP2 promotes osteogenic differentiation by activating transcription factors such as Runx2 [65], which then binds to promoters of osteogenic genes to regulate bone turnover [66]. In this study, Se treatment attenuated the decreased expression of BMP2 and Runx2 in GIOP rats. These results suggest that Se has a bone protective effect in GIOP rats by promoting the expression of OPG, OCN, BMP2 and Runx2, and inhibiting the expression of RANKL and CTX.

MDA is a product of peroxidation reactions that reflects oxidative stress. SOD and GSH are two antioxidant enzymes that can resist and repair oxygen-free radicals-induced damage to cells [67]. Here, Se could reduce MDA and increase GSH and SOD activities in GIOP rats and MC3T3-E1 cells. These results suggest that Se has antioxidant stress effects in vivo and in vitro GIOP models.

However, this study should also acknowledge its limitations. First of all, because no specific questionnaire was set, we did not screen out GIOP or PMOP patients in the final analysis. The study only discussed the relationship between dietary Se intake and OP prevalence, without further discussing that between dietary Se intake and the prevalence of GIOP or PMOP. Secondly, a compact RA system was used in this study to detect BMD of the phalanges, whereas the gold diagnosis standard for OP is hip and spine BMD measurements using DEXA [68, 69]. DEXA diagnostics are expensive and require frequent calibration, so they are still rarely used in developing countries. In fact, several cohort studies have studied the efficacy of phalangeal rheumatoid arthritis using the same measurement system [23, 70]. BMD tests were performed on the middle phalangeal bones, lumbar spine (L2-L4), and total hip joint in 221 women (50–75 years). The results found that compared with DEXA, RA has a sensitivity of 82.9% to identify OP and a negative predictive value of 90% [71]. Third, it may be difficult to infer Se intake from SFFQ, because Se content in food varies greatly [72]. Meanwhile, Se is affected by food preparation or cooking [73] and varies largely [74]. However, SFFQ is utilized to evaluate dietary Se intake and is effective and reliable for estimating Se intake [75, 76]. Fourthly, the study only preliminarily verified the effects of Se on bone protection and antioxidant stress in GIOP models, without further exploring the specific downstream regulatory mechanisms. It is hoped that the specific mechanisms of Se in preventing GIOP can be further explored in future studies.

## Conclusion

Participants with lower dietary Se intake had a higher OP prevalence. Se can effectively weaken GIOP through bone protection and anti-oxidative stress, and is a promising candidate for GIOP therapy.

## Data Availability

The datasets used and/or analyzed during the present study are available from the corresponding author on reasonable request.
