# Correction: Association between dietary selenium intake and the prevalence of osteoporosis and its role in the treatment of glucocorticoid-induced osteoporosis

**DOI:** 10.1186/s13018-023-04500-2

**Published:** 2024-01-05

**Authors:** Yi Luo, Yaolin Xiang, Banghua Lu, Xiaoyan Tan, Yanqiong Li, HuiHui Mao, Qin Huang

**Affiliations:** 1 Department of Nephropathy and Rheumatology, The Central Hospital of Enshi Tujia and Miao Autonomous Prefecture, No. 158, Wuyang Avenue, Enshi City, 445099 Hubei Province China; 2 Department of Neonatology, Renmin Hospital Afliated to Hubei University for Nationalities, Enshi City, 445099 Hubei Province China

**Correction: Journal of Orthopaedic Surgery and Research** 10.1186/s13018-023-04276-5

Following publication of the original article [1], the author reported that the author HuiHui Mao was omitted from the author group. HuiHui Mao has been added to the author group and are presented correctly in this correction article.

The original article [1] has been corrected.
